# HIV sexual transmission risks in the context of clinical care: a prospective study of behavioural correlates of HIV suppression in a community sample, Atlanta, GA, USA

**DOI:** 10.7448/IAS.18.1.19930

**Published:** 2015-08-05

**Authors:** Seth C Kalichman, Chauncey Cherry, Moira O Kalichman, Christopher Washington, Tamar Grebler, Cindy Merely, Brandi Welles, Jennifer Pellowski, Christopher Kegler

**Affiliations:** Department of Psychology and Center for Health Intervention and Prevention, University of Connecticut, Storrs, CT, USA

**Keywords:** HIV prevention, treatment as prevention, sexual HIV transmission, HIV suppression

## Abstract

**Introduction:**

Antiretroviral therapy (ART) improves the health of people living with HIV and has the potential to reduce HIV infectiousness, thereby preventing HIV transmission. However, the success of ART for HIV prevention hinges on sustained ART adherence and avoiding sexually transmitted infections (STI).

**Objectives:**

To determine the sexual behaviours and HIV transmission risks of individuals with suppressed and unsuppressed HIV replication (i.e., viral load).

**Methods:**

Assessed HIV sexual transmission risks among individuals with clinically determined suppressed and unsuppressed HIV. Participants were 760 men and 280 women living with HIV in Atlanta, GA, USA, who completed behavioural assessments, 28-daily prospective sexual behaviour diaries, one-month prospective unannounced pill counts for ART adherence, urine screening for illicit drug use and medical record chart abstraction for HIV viral load.

**Results:**

Individuals with unsuppressed HIV demonstrated a constellation of behavioural risks for transmitting HIV to uninfected sex partners that included symptoms of STI and substance use. In addition, 15% of participants with suppressed HIV had recent STI symptoms/diagnoses, indicating significant risks for sexual infectiousness despite their HIV suppression in blood plasma. Overall, 38% of participants were at risk for elevated sexual infectiousness and just as many engaged in unprotected sexual intercourse with non-HIV-infected partners.

**Conclusions:**

Implementation strategies for using HIV treatments as HIV prevention requires enhanced behavioural interventions that extend beyond ART to address substance use and sexual health that will otherwise undermine the potential preventive impact of early ART.

## Introduction

Antiretroviral therapies (ART) effectively suppress HIV replication and can restore immune function to improve the health of HIV-infected persons. These medications are also now central to HIV prevention. It has long been shown that HIV suppression in blood plasma is associated with reduced risks for sexual transmission of HIV [1]. There is compelling evidence that early treatment with ART reduces HIV transmission risks when individuals are adherent to their medication regimen and when they are cleared of any co-occurring sexually transmitted infections (STI) [2]. Clinical guidelines are, therefore, emerging for early treatment with ART as an HIV prevention strategy in clinical settings [3].

The success of ART for HIV prevention hinges on: (a) maintaining ART adherence to sustain viral suppression [4] and (b) avoiding STI and other sources of genital tract inflammation. Even when ART adherence is high and blood plasma viral suppression is maintained, individuals with genital tract inflammation are highly infectious [5–7]. Research has shown that there is a dose-relationship between leucocyte activity in the genital tract and HIV shedding [8]. In one prospective study, detection of genital tract HIV RNA increased 1.36 for every 1000 cell increase in genital tract leucocytes [9]. Past research has also shown that urethritis is associated with an eightfold increase in HIV in the seminal plasma compartment [10]. A review of 19 studies that examined the relationship between blood plasma and semen viral load found an average correlation of only *r*=0.44 [11]. Viral shedding in the genital tract that results from co-occurring STI may, therefore, at least in part account for the discordance between HIV in blood plasma and HIV in genital fluids.

HIV sexual infectiousness should, therefore, be conceptualized within a broader context of behaviour and sexual health. Specifically, increased exposure to sexually transmitted pathogens that occur for individuals who engage in higher rates of unprotected sex with greater numbers of partners ultimately means that those persons who are most sexually active may be most infectious. Also of importance are behaviours that influence both ART adherence and sexual risk practices. Of particular importance are alcohol and drug use, both of which are robust predictors of ART adherence and unprotected sex in people living with HIV. Studies show that even moderate alcohol use is associated with poor ART adherence [12,13]. Similarly, alcohol and other drugs are among the most reliable predictors of HIV transmission risk behaviours in people living with HIV [14,15]. In one study of people living with HIV who drink alcohol, one in three were not virally suppressed and one in five had been diagnosed with a recent STI [16]. Substance use, therefore, has the potential to undermine the preventive effects of HIV treatment by both impeding ART adherence and increasing risks for exposure to STI [17].

In the current study, we examined the sexual behaviour and sexual health context of HIV suppression among people living with HIV. To achieve prevention goals of HIV treatment, behavioural factors associated with unsuppressed HIV replication must be identified and addressed. Our study aims are, therefore, focused on sexual risks for HIV transmission among persons who are HIV suppressed in comparison to persons who are not viral suppressed. Because viral suppression is a function of behaviour, namely ART adherence, we predicted that individuals with unsuppressed HIV would be characterized by a constellation of co-occurring behavioural risk factors, including alcohol and drug use as well as high-risk sexual behaviours. We, therefore, tested the hypothesis that individuals with unsuppressed HIV will demonstrate parallel behavioural risks for transmitting HIV to uninfected sex partners as well as active substance use and unprotected sex with HIV negative and unknown HIV status (i.e., non-concordant) sex partners.

## Methods

### Participants

Participants were 760 men and 280 women recruited from community services and infectious disease clinics during a 12-month period between 2013 and 2014. The study was conducted in Atlanta, GA, USA, with an annual incidence of 30.3 per 100,000, exceeding the 19.6 per 100,000-population rate of HIV in major US cities. Eligible participants were aged 18 or older and HIV-positive.

### Measures

Participants provided five sources of data: (a) audio-computer-assisted self-interviews (ACASI) to assess demographic and health characteristics; (b) urine specimens for substance use screening; (c) unannounced pill counts to assess ART adherence; (d) daily text-message sexual behaviour diaries and (e) HIV RNA (viral load) and CD4 cell counts from medical records. The specific measures are described below.

#### Computer-assisted interviews for demographic and health characteristics

Participants were asked their gender, age, years of education, income, ethnicity, employment status and the year that they first tested HIV positive. Participants indicated whether they were currently taking ART and whether they knew the results of their most recent HIV viral load test. For those who indicated knowing their viral load, we asked whether their most recent viral load was undetectable or detectable. The three-item Alcohol Use Disorder Identification Test consumption subscale (AUDIT-C) was used to assess the quantity and frequency of alcohol use. The AUDIT-C is a modification of the 10-item AUDIT that reliably identifies people who are hazardous drinkers [18]. We adapted established methods of assessing recent symptoms of an STI and STI diagnoses [19]. Specifically, participants reported whether they had experienced three common STI symptoms (genital discharge, genital pain and ulceration) and whether they had been diagnosed with any of eight non-HIV STI or sources of genital disease during the past three months.

#### Urine screening for substance use

To screen for illicit drug use, we conducted a multi-panel urine dip-test. This test strip uses a lateral flow chromatographic immunoassay for qualitative detection of 12 drugs and drug metabolites (Redwood Toxicology Labs – Reditest-12). These tests are FDA approved and are reliable and valid for initial drug screening.

#### Unannounced pill count adherence

Participants consented to three unannounced pill counts that occurred over a six-week period. Unannounced pill counts are reliable and valid in assessing medication adherence when conducted in homes [20] and on cell-phones [21,22]. In this study, we conducted unannounced cell-phone-based pill counts using study-provided cell phones. Following the initial office-based interview that included a full accounting of all prescription medications and training in the pill counting procedure, participants were called at three unscheduled times over 12–16 day intervals. The first of the three pill counts is used to establish the number of pills in possession with the subsequent two pill counts allowing for calculation of adherence, defined as the ratio of pills counted relative to pills prescribed, taking into account the number of pills dispensed. Adherence was examined using three clinically meaningful levels: 75, 85 and 95% of medications taken [4].

#### Sexual behaviour text-message diaries

We used an interactive text-diary assessment to collect daily data on sexual behaviours. Participants were instructed in the use of text message functions of their study-provided cell phone. Brief daily assessments were designed and delivered using interactive short message system response. Electronic diaries have provided reliable data collection of socially sensitive behaviours [23,24]. Participants received a text-prompt to initiate and answer questions about their sexual activity during the previous day. The questions specifically asked about whether participants had sex yesterday and if so, whether they engaged in vaginal and anal intercourse with and without condoms, if substance use occurred in the context of sex and whether their partner was aware of their HIV status and their knowledge of their partner's HIV status. Daily sexual behaviour data were recorded by entering numerical responses using the cell phone keypad. The data were stored on a secured central server. Sexual behaviour assessments were administered prospectively for 28 consecutive days following the initial office assessment.

#### Chart abstracted viral load and CD4 cell counts

We used a participant-assisted method for collecting chart abstracted viral load and CD4 cell counts from participants’ medical records. Participants were given a form that requested their doctor's office to provide results and dates of their most recent, and not older than three months, viral load and CD4 cell counts. These data were, therefore, obtained directly by the participant from their HIV care provider. The form included a place for the provider's office stamp or signature to assure data authenticity. HIV RNA below detection was defined as less than 100 copies/mL for uniformity across providers.

## Procedures

Men and women living with HIV were recruited through targeted community sampling with both venue recruitment and snowball sampling techniques. Venue recruitment relied on responses to brochures placed in waiting rooms of HIV service providers and infectious disease clinics. Study brochures were placed in clinic waiting rooms throughout the Atlanta metropolitan area. More than 60% of participants were recruited from a major, multi-service HIV clinic and academic outpatient centre. The remaining participants were receiving health care from a mix of public (county) and private medical providers.

At the initial office assessment session, participants were provided with informed consent and completed a computer-assisted interview to collect demographic and health information. We used computerized interviews because they have been shown to increase responses to socially sensitive measures [25,26]. Participants were trained to conduct phone-based unannounced pill counts and trained in the daily cell-phone text message assessments. In addition, participants provided a urine sample for drug screening. We also asked participants to obtain their most recent HIV viral load and CD4 cell count results from their medical provider. Participants then completed three unannounced phone-based pill counts to determine ART adherence over the next month and completed 28 prospective daily text message surveys to assess sexual activity. At the end of the one-month observation period, participants returned their chart abstracted viral load and CD4 cell count data to the research office. Participants were reimbursed $145 for completing all measures and providing all data. The university Institutional Review Board approved all procedures.

## Data analyses

We constructed logistic regression models to test the associations between HIV viral load (i.e., suppressed HIV vs. unsuppressed HIV) and participant demographic, health, substance use and sexual behaviour characteristics. Bivariate regressions with unadjusted odds ratios were initially performed followed by multivariable models that adjusted for non-redundant variables found significant in bivariate analyses. To test our main study hypothesis that unsuppressed HIV would be associated with substance use and sexual transmission risk behaviours, we constructed three multivariable models where HIV suppressed and unsuppressed groups were compared on: (a) STI diagnoses and symptoms experienced in the previous three months, (b) engaging in any vaginal or anal intercourse without condoms in the prospective 28-days and (c) engaging in any vaginal or anal intercourse without condoms with HIV non-concordant sex partners in the prospective 28 days. Because 77 participants were not currently receiving HIV treatment, we repeated analyses excluding these participants. In addition, we examined gender effects by repeating the models separately for men and women. For all analyses, statistical significance was defined by *p*<0.05.

## Results

Chart abstracted viral load results showed that 289 (27%) participants had detectable (unsuppressed) HIV in blood plasma. Table 1 shows the demographic characteristics of individuals with suppressed and unsuppressed HIV. Participants with unsuppressed HIV were more likely male and transgender, had lower incomes, were more likely unemployed, were significantly younger and had been diagnosed with HIV for fewer years than their counterparts with suppressed HIV.

**Table 1:** Demographic characteristics of people living with HIV who had suppressed and unsuppressed HIV viral loads

	Suppressed HIV (*n=*751)		Unsuppressed HIV (*n=*289)			
				
Characteristic	*N*	%	*N*	%	OR	95% CI
Gender						
Men	534	71	226	78		
Women	218	29	62	22	0.68**	0.49–0.94
Transgender	35	5	29	10	2.28**	1.37–3.81
Race						
African–American	683	91	267	92		
Caucasian	50	7	11	4		
Other races	20	2	11	4	1.21	0.92–1.60
Income<$10,000	466	62	210	73	0.61**	0.45–0.82
Employment						
Unemployed	185	25	125	43		
Employed	105	14	43	15		
Disabled	432	57	111	38		
Student	30	4	10	3	0.65**	0.55–0.75
	M	SD	M	SD		
Age	48.1	9.6	42.24	9.83	0.94**	0.93–0.95
Education	12.6	1.8	12.5	13.9	0.95	0.89–1.03
Years since testing HIV positive	14.9	7.9	12.2	8.5	0.95*	0.94–0.97

** *p*<0.01;

* *p*<0.05.

### HIV-related health status

Participants with unsuppressed HIV evidenced poorer immune functioning, with a greater likelihood of CD4 cell counts below 200 cell/ml (see Table 2). Individuals with unsuppressed HIV were less likely to receive ART, and those who were treated with ART had significantly poorer adherence; 46% of unsuppressed participants were <85% adherent and 31% were <75% adherent. In addition, individuals who had unsuppressed HIV were significantly more likely to not know their current viral load, and among those who stated they knew their most recent viral load, one in three incorrectly believed that their viral load was suppressed.

**Table 2:** HIV-related health and sexual health characteristics of people living with HIV who had suppressed and unsuppressed HIV viral loads

	Suppressed HIV (*n=*751)		Unsuppressed HIV (*n=*289)			
				
	*N*	%	*N*	%	OR	95% CI
CD4<200	77	10	98	35	4.63**	3.30–6.51
Currently receiving ART	743	98	221	76	0.04**	0.02–0.08
Knowledge of viral load						
Do not know viral load	283	38	178	62	2.66**	2.01–3.52
Believe HIV is unsuppressed	31	6	72	65		
Believe HIV is suppressed	439	94	38	35	26.38**	15.70–45.85
ART adherence						
Mean (SD) adherence	86.9	15.8	80.2	21.7	0.15**	0.06–0.32
<95%	303	43	73	35	0.71*	0.51–0.97
<85%	220	31	96	46	0.53**	0.39–0.73
<75%	128	18	66	31	0.48**	0.34–0.68
STI symptoms						
Genital discharge	43	6	32	11	2.05**	1.27–3.31
Genital pain	43	6	36	13	2.35**	1.47–3.74
Genital ulcer	50	7	30	10	1.62*	1.01–2.61
Any STI symptom	104	14	70	24	1.99**	1.42–2.80
STI diagnoses						
Gonorrhoea	17	2	13	5		
Chlamydia	14	2	9	5		
Syphilis	28	4	25	9		
HSV	62	8	29	10		
NGU	5	1	4	1		
Trichamoniasis	10	1	6	2		
Any STI diagnosis	110	15	71	25	1.90**	1.36–2.66

** *p*<0.01;

* *p*<0.05.

### Sexual health

Table 2 shows the sexual health of HIV-suppressed and unsuppressed groups. Individuals with unsuppressed HIV were significantly more likely to have recently experienced STI symptoms, including genital discharge, pain and genital ulcers. In addition, unsuppressed HIV was associated with having been diagnosed with an STI in the previous three months; 25% of participants with unsuppressed HIV had a new STI compared to 15% of participants with suppressed HIV.

### Substance use

Results showed that unsuppressed HIV was associated with a greater likelihood of current alcohol use, greater quantity of alcohol consumption, a trend towards more frequent binge drinking (see Table 3). More than half of the sample screened positive for any active non-alcohol drug use detected in urine. The most common drugs detected were THC and cocaine. Although there was no association between THC detection and HIV viral suppression, individuals with unsuppressed HIV were significantly more likely to screen positive for cocaine use than their viral suppressed counterparts, with 32 and 23% of viral unsuppressed and suppressed persons testing positive for cocaine, respectively.

**Table 3:** Substance use among people living with HIV who had suppressed and unsuppressed HIV viral loads

	Suppressed HIV (*n=*752)		Unsuppressed HIV (*n=*289)			
				
	*N*	%	*N*	%	OR	95% CI
Alcohol use frequency						
Never	358	48	111	38		
Monthly	130	17	60	21		
2–4 times/month	157	21	69	24		
2–3 times/week	78	10	35	12		
4+ times/week	26	4	14	5	1.14*	1.02–1.27
Alcohol quantity						
1–2 drinks	257	34	110	38		
3–4 drinks	102	14	50	17		
5–6 drinks	17	2	5	2		
7–9 drinks	8	1	8	3		
10+ drinks	7	1	5	2	0.93*	0.88–0.99
Consumes 6+ drinks						
Never	235	31	88	31		
<Monthly	79	11	44	15		
Monthly	22	3	19	7		
Weekly	44	6	20	7		
Almost daily/daily	11	2	7	2	0.94+	0.89–1.00
Drug use						
Cocaine use	171	23	90	32	1.57*	1.16–2.21
THC use	222	30	80	28	0.93	0.68–1.26
Any drug use	395	53	162	57	1.19	0.90–1.57

** *p*<0.01;

* *p*<0.05;

+ *p*<0.10.

### Sexual transmission risk behaviours

Individuals with unsuppressed HIV were significantly more likely to engage in an array of sexual transmission risk behaviours including unprotected vaginal intercourse and unprotected anal intercourse. Unsuppressed HIV was associated with engaging in sex without condoms for a greater number of the subsequent 28 days of observation (see Table 4). Individuals with unsuppressed HIV reported unprotected intercourse on significantly more days with both HIV concordant and HIV non-concordant sex partners and were more likely to report unprotected sexual activity that co-occurred with substance use. Finally, more than one in four participants indicated that they engaged in sex without condoms with a partner that they had not disclosed their HIV status.

**Table 4:** Sexual behaviours over 28-prospective days among people living with HIV who had suppressed and unsuppressed HIV viral loads

	Suppressed HIV (*n=*752)		Unsuppressed HIV (*n=*289)			
				
Behaviour	*N*	%	*N*	%	OR	95% CI
Vaginal sex w/o condoms	269	35	131	45	1.49**	1.13–1.97
Condom protected vaginal sex	248	32	99	34	1.06	0.80–1.41
Anal sex w/o condoms	278	36	132	64	1.44**	1.09–1.89
Condom protected anal sex	258	34	119	41	1.34*	1.02–1.78
Any unprotected vaginal/anal sex	389	51	174	60	1.42**	1.08–1.87
Days any sex w/o condoms						
0	366	48	115	40		
1	166	22	59	20		
2–3	112	14	47	16		
4+	111	14	68	24	1.07**	1.02–1.11
Days concordant sex w/o condoms	Ref					
0	562	75	184	64		
1	107	14	50	17		
2–3	50	6	29	10		
4+	36	5	26	9	1.09**	1.01–1.17
Days non-concordant sex w/o condoms						
0	477	63	165	57		
1	130	17	51	18		
2–3	85	11	41	14		
4+	63	8	32	11	1.06*	1.01–1.11
Substance use–sex w/o condoms	140	38	86	51	1.08*	1.01–1.15
Undisclosed HIV status w/o condoms	180	24	85	29	1.06	0.99–1.09

** *p*<0.01;

* *p*<0.05.

### Multivariable models

**Figure 1:**
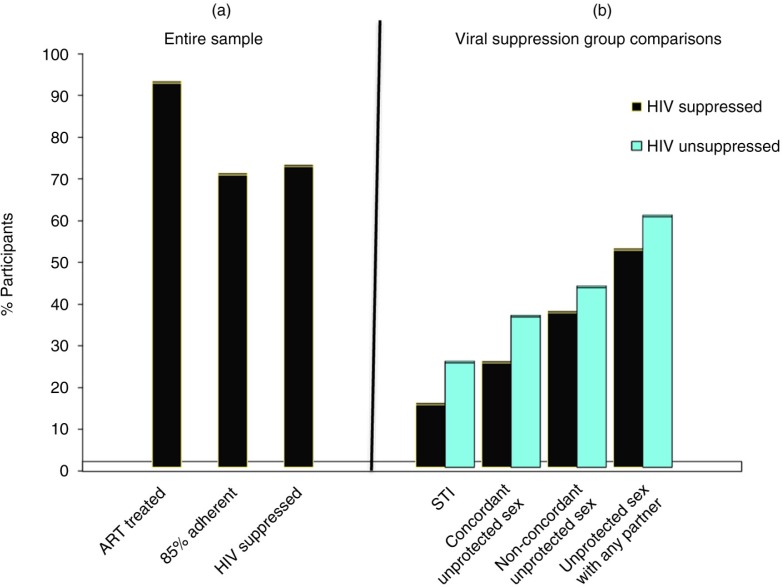
(a) Percent of participants receiving ART, ART adherent and achieving HIV suppression and (b) sexual HIV transmission risks among individuals with suppressed and unsuppressed HIV.

Figure 1a summarizes the proportions of the sample that were being treated with ART, at least 85% adherent and viral suppressed, indicating that one in four participants were likely sexually infectious based on blood plasma viral load alone. Figure 1b shows the differences in recent STI symptoms/diagnoses, and engaging in sexual intercourse without condoms in HIV concordant and non-concordant relationships, and condomless sex with any partners. Three multivariable logistic regression models tested the independent association of HIV viral suppression and HIV transmission risks from: (a) having recent STI diagnosis/STI symptoms, (b) engaging in any condomless intercourse and (c) engaging in condomless sex with HIV non-concordant partners. All models are controlled for participant age, years since testing HIV positive, gender, transsexual identity, income, employment status, CD4 cell count, being treated with ART and knowledge of most recent viral load. Results of adjusted models showed that poorer HIV suppression was related to having recent STI, adjusted odds ratio (OR)=1.59, *p*<0.01, 95% confidence interval (CI) 1.12–2.25; engaging in more condomless intercourse over the prospective 28 days, adj OR=1.06, *p*<0.05, 95% CI 1.01–1.10 and engaging in more condomless sex with non-concordant partners, adj OR=1.06, *p*=0.08, 0.99–1.12.

### Potential impact of untreated participants

We repeated the multivariable models for the 964 participants receiving ART. Results showed the same pattern as observed for the full sample. In these models, poorer HIV suppression was related to having recent STI, adj OR=1.52, *p*<0.05, 95% CI 1.05–2.19, and engaging in more condomless intercourse, adj OR=1.05, *p*<0.06, 95% CI 1.00–1.10. For participants receiving ART, the multivariable model for engaging in more condomless sex with non-concordant partners was also non-significant, adj OR=0.83, *p*>0.10, 95% CI 0.55–1.22.

### Gender analyses

We examined the key models in this study for only the 280 women. Results of bivariate models showed that poorer HIV suppression was associated with greater STI symptoms, adj OR=1.92, *p*<0.05, 95% CI 1.03–3.56, and STI diagnoses, adj OR=1.83, *p*<0.06, 95% CI 0.96–3.50. However, results from the multivariable models showed different patterns of results than observed in the full sample. For women, HIV suppression was not related to having recent STI in the multivariable model, adj OR=1.16, *p*>0.10, 95% CI 0.58–2.32, nor was viral suppression related to engaging in condomless intercourse, adj OR=1.03, *p*>0.10, 95% CI 0.95–1.14. However, for women, the multivariable model for engaging in condomless sex with non-concordant partners showed that poorer viral suppression was related to engaging in greater non-concordant sex, adj OR=0.38, *p*<0.05, 95% CI 0.16–0.92. Thus, interpretation of the study findings should take gender into consideration.

## Discussion

Results of the current study found that more than one in four people living with HIV recruited from community and clinical services in a city with a large and growing population of people living with HIV had unsuppressed HIV replication. Unsuppressed HIV was more common among younger unemployed and lower income men who had been diagnosed for fewer years than those with suppressed virus. As expected, unsuppressed HIV was associated with poorer ART adherence and lower CD4 cell counts. In addition, individuals who had unsuppressed HIV were significantly less likely to know their HIV viral load, and among persons with unsuppressed HIV who stated they knew their viral load, one in three incorrectly believed their viral load was suppressed.

Our study confirmed our main hypothesis that individuals with unsuppressed HIV would demonstrate a constellation of behavioural risks for transmitting HIV to uninfected sex partners. Individuals with unsuppressed HIV engaged in higher rates of unprotected sex with non-concordant partners, had higher rates of STI symptoms and diagnoses, and more substance use including substance use in the context of sex. Multivariable models demonstrated that the increased risks observed among unsuppressed participants were not accounted for by potential confounding from other variables measured. Participants with unsuppressed HIV were also least aware and most likely mistaken of their HIV viral load. Taken together, our results show that one in four persons were at risk for declining health and onward HIV transmission.

We also found that 15% of HIV-suppressed participants had recent STI symptoms or a recent STI diagnoses, indicating significant risks for sexual infectiousness despite their HIV suppression in blood plasma. It was common for viral-suppressed participants to engage in sexual intercourse without condoms with HIV non-concordant partners over the one-month observation period. In addition, more than half of viral-suppressed participants screened positive for active drug use and nearly one in four had not disclosed their HIV status to their current sex partners. Thus, even in the context of ART adherence and successful viral suppression, a critical mass of our sample was at risk for increased infectiousness and potential HIV transmission to uninfected partners. While these participants fall within the scope of what may appear to be the more optimistic side of the HIV care continuum in terms of accessing HIV treatments for prevention [27], they are at risk of heightened infectiousness due to non-adherence, active substance and HIV transmission by virtue of prevalent STI symptoms and diagnoses.

Subsequent analyses found that the overall pattern of results was unaffected by including persons who were not currently receiving ART. These individuals were, of course, concentrated among those with unsuppressed HIV. Nevertheless, results did not vary when these persons were excluded. In contrast, our examination of potential gender effects in the study was significant. The pattern of results for multivariable models for women were strikingly different from the overall male-dominated sample. For women, viral suppression was not associated with recent STI and practicing condomless sex. However, unlike the sample as a whole, poorer viral suppression was related to engaging in greater non-concordant condomless sex. Our sample size of women was relatively small and disproportionate, limiting our ability to include participant gender as a factor in the analyses. The apparent difference in the association between viral suppression and sexual transmission risks in women do caution against generalizing the study findings across genders and indicate the need to replicate this study with a larger sample of women.

These findings should be interpreted in light of the study limitations. We relied on a convenience sample that cannot be considered representative of men living with HIV infection. The sample also came from a wide range of providers that likely varied in sexual health services including STI screening and treatment. Our findings are constrained by being conducted in the southeastern United States. The setting represents a large urban area with a major medical centre that includes a multi-service HIV clinic. Thus, our findings are likely of limited generalizability to settings with more constrained healthcare resources. The study is also limited by a small number of women, especially given then evidence that the observed findings differ for women. The study also relied on self-report instruments to assess sexual behaviours. Although we collected sexual behaviours using state of the science-daily assessments, these data may still be subject to reporting biases. Socially sensitive behaviours assessed by self-report may be underreported, suggesting that rates of unprotected sex, HIV status disclosure, STI symptoms and diagnoses in this study should be considered lower bound estimates, or best case scenarios. In addition, we do not know the prevalence of ART resistance in our sample or the broader population, and we do not know how relative rates of resistance would alter our findings. With these limitations in mind, we believe that our study findings have implications for implementing early HIV treatment as an HIV prevention strategy.

The prevention benefits of ART will not be realized for persons who are viral unsuppressed or for those who are viral suppressed and experience HIV shedding as a result of genital tract inflammation. In this study, 38% of the sample can be considered likely sexually infectious – unsuppressed HIV or suppressed with STI symptoms/diagnoses – and just as many engaged in sex without condoms with an HIV non-concordant partner. Behavioural interventions that aim to sustain ART adherence and improve the sexual heath of people with HIV should, therefore, be a priority in the implementation of early treatment for prevention [3]. The most useful interventions will be those that are crafted to fit clinical settings and address active substance use as an undermining factor of adherence and sexual risks [28–30].

## Conclusions

Implementation strategies for using HIV treatments as HIV prevention requires enhanced behavioural interventions that extend beyond ART to address substance use and sexual health that will otherwise undermine the potential preventive impact of early ART. These strategies may also vary for men and women. Because not every person on ART will require enhanced behavioural interventions, clinical services should incorporate sexual behaviour and substance use assessments, STI screening and tests for genital tract inflammation to triage patients for targeted interventions. Assuring the success of early ART for HIV prevention will require investing in optimal behavioural interventions that take full advantage of the opportunities offered by early HIV treatments.
